# Potato and warm moist compresses to accelerate healing of limb swelling and bruising after venous stent placement: A case report

**DOI:** 10.1097/MD.0000000000051049

**Published:** 2026-10-09

**Authors:** Qin Zhang, Zhuoyin Wang, Hongqun Zhang, Xiaojuan Han, Jiani Bao, Yaru Yang, Qi Chen

**Affiliations:** a Department of Blood Purification Center, Ningbo Beilun Third People’s Hospital, Ningbo, Zhejiang, China; b Department of Surgery, Ningbo Beilun Third People’s Hospital, Ningbo, Zhejiang, China; c Department of Nursing Department, Ningbo Beilun Third People’s Hospital, Ningbo, Zhejiang, China; d Department of ICU, Ningbo Beilun Third People’s Hospital, Ningbo, Zhejiang, China; e Department of Surgery, Ningbo Mingzhou Hospital, Ningbo, Zhejiang, China.

**Keywords:** AVF, maintenance hemodialysis, narrower, potato, swelling, warm moist compresses

## Abstract

**Rationale:**

Hemodialysis is an essential renal replacement therapy for patients with end-stage renal disease, and an autologous arteriovenous fistula (AVF) is preferred because of its durability and low complication rate. Maintaining AVF patency is critical for effective dialysis. AVF stenosis may reduce blood flow, impair dialysis adequacy, and compromise vascular access function. Digital subtraction angiography-guided angioplasty and venous stent placement are commonly used to restore patency. Postoperative swelling, bruising, pain, and restricted limb movement may occur and delay recovery. This report describes the use of fresh potato slices combined with warm moist compresses after venous stent placement.

**Patient concerns:**

A man in his 70s with stage 5 chronic kidney disease had received maintenance hemodialysis through a right forearm AVF for 5 years. Persistently elevated venous pressure prompted imaging, which revealed AVF outflow vein stenosis. He underwent digital subtraction angiography-guided venous stent placement. Marked swelling, extensive bruising, pain, numbness, and reduced joint mobility subsequently developed in the right upper extremity.

**Diagnoses:**

AVF stenosis, stage 5 chronic kidney disease, maintenance hemodialysis, renal anemia, type 2 diabetes mellitus, and hypertension.

**Interventions:**

In addition to routine postoperative nursing care, fresh potato slices were applied along the affected vascular pathway for 2 hours, followed by a warm moist compress at 40°C to 45°C for 20 to 30 minutes. Treatment was administered 3 times daily.

**Outcomes:**

The pain score decreased from 6 to 3 after treatment and reached 0 by postoperative day 4. Swelling and bruising substantially subsided, and joint mobility approached normal. By postoperative day 6, bruising had further diminished, limb movement was normal, and the AVF could be punctured routinely. No infection, skin allergy, irritant erythema, or other intervention-related adverse events were observed.

**Lessons:**

Fresh potato slices combined with warm moist compresses were associated with improvement in postoperative swelling, bruising, pain, and limb mobility. The intervention was simple, inexpensive, and well tolerated. Because this was a single case and comprehensive nursing care was provided concurrently, its independent effect cannot be determined. Larger prospective controlled studies using standardized quantitative assessments are needed before routine clinical use.

## 1. Introduction

End-stage renal disease has become a major global public health concern, and its prevalence continues to increase worldwide.^[1–3]^ Maintenance hemodialysis (MHD) is a principal renal replacement therapy for patients with end-stage renal disease.^[4–7]^ Reliable vascular access is essential for the delivery of effective hemodialysis.^[8,9]^ An arteriovenous fistula (AVF) is the preferred form of vascular access because of its relatively low complication rate and long-term patency.^[10–13]^ Despite these advantages, AVFs remain susceptible to stenosis during long-term use. Stenosis is a major cause of AVF dysfunction and access failure. AVF stenosis may cause upper-extremity swelling and venous hypertension, thereby compromising dialysis adequacy and patients’ quality of life.^[12,14]^

Digital subtraction angiography (DSA)-guided balloon angioplasty, with stent placement when indicated, is an established endovascular treatment for AVF stenosis.^[15–19]^ This endovascular approach is minimally invasive and generally considered safe. Nevertheless, puncture-related complications may occur, including local hematoma, tissue edema, and subcutaneous ecchymosis. These complications may cause pain and discomfort, prolong hospitalization, and increase patient anxiety. In severe cases, they may also impair AVF function or interfere with subsequent vascular access cannulation.

Standardized management protocols for these postoperative complications remain lacking in clinical practice. Conventional nursing measures include compression bandaging, elevation of the affected limb, and local warm moist compresses. Among these measures, warm moist compresses are widely used in routine clinical care.^[20]^ By increasing local tissue temperature and circulation, warm moist compresses may facilitate fluid redistribution and the resolution of localized swelling. Their effects may be limited or slow to develop, particularly in older adults, patients with impaired vascular function, and those receiving anticoagulant therapy. Potatoes contain starch, vitamins, carotenoids, glycoalkaloids such as solanine, and several minerals. Some potato-derived constituents have been reported to exhibit antioxidant and anti-inflammatory activities, although their clinical effects when applied topically remain uncertain. In this patient, the application of fresh potato slices combined with warm moist compresses was followed by a visible reduction in swelling and bruising of the affected limb after DSA-guided stent placement. This inexpensive and readily available approach may represent a feasible adjunctive nursing intervention, although its effectiveness and safety require confirmation in controlled studies.

## 2. Case presentation

A man in his 70s with stage 5 chronic kidney disease secondary to hypertensive nephropathy had been receiving MHD for 5 years. Hemodialysis was performed through a right forearm AVF, which had functioned without notable complications. During the month before admission, elevated venous pressure was consistently observed during hemodialysis, whereas arterial pressure remained within the normal range. Potential procedural causes, including inadequate anticoagulation and kinking or twisting of the dialysis tubing, were excluded. Subsequent computed tomography angiography of the right upper extremity revealed stenosis within the AVF circuit (Fig. 1). To further evaluate and treat the stenosis, the patient underwent endovascular stent placement under local anesthesia and DSA guidance. The procedure was completed successfully, and the patient returned to the ward in stable condition.

**Figure 1.**
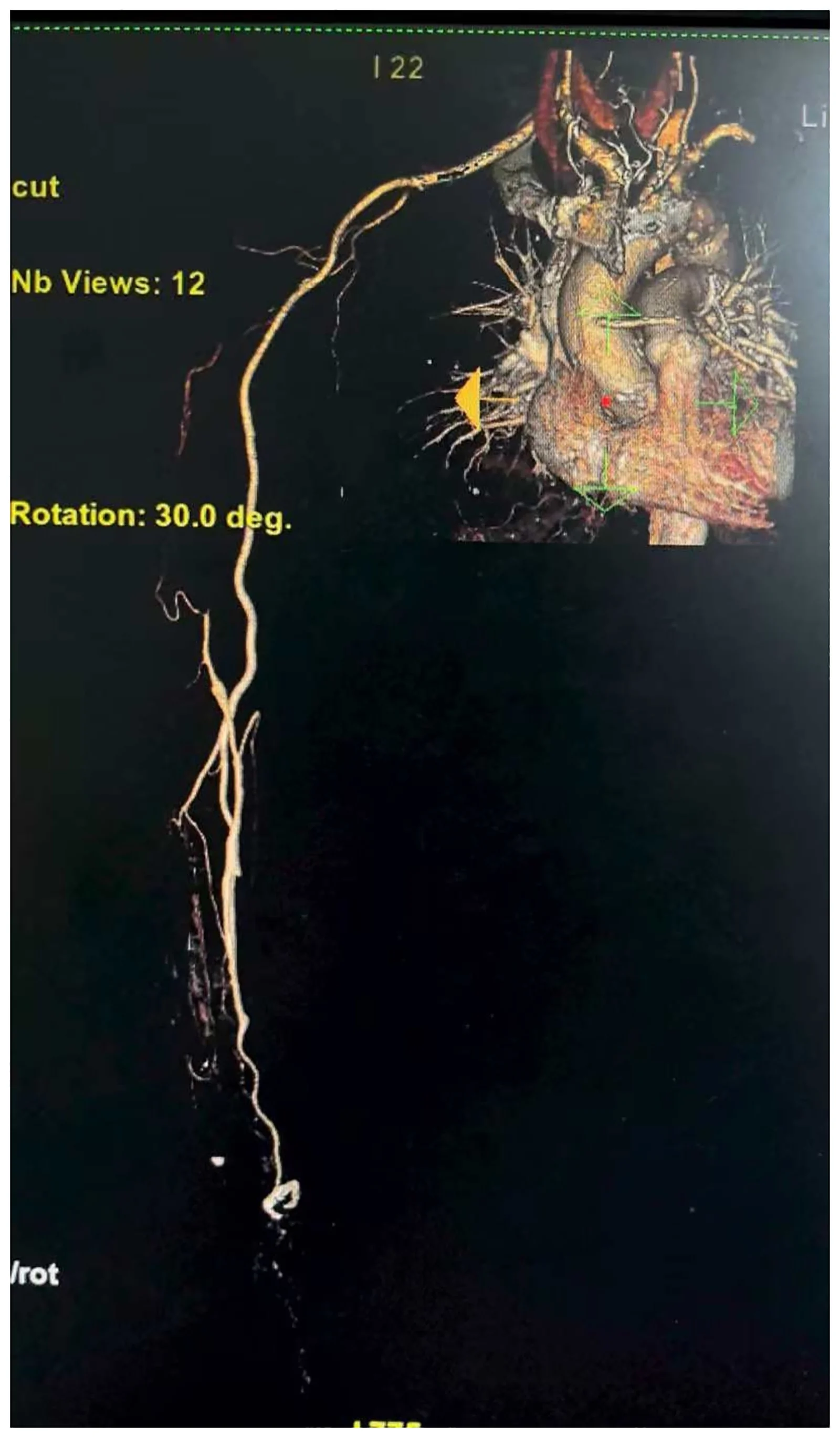
Computed tomography angiography imaging results.

On the day of the procedure, the patient reported swelling and pain in the right upper extremity, with a numeric rating scale (NRS) pain score of 3. By postoperative day 1, marked swelling of the right upper arm had developed, accompanied by skin tightness and patchy purplish ecchymosis (Fig. 2). The NRS pain score increased to 6. The patient also reported numbness of the affected limb and reduced mobility of the adjacent joints.

**Figure 2.**
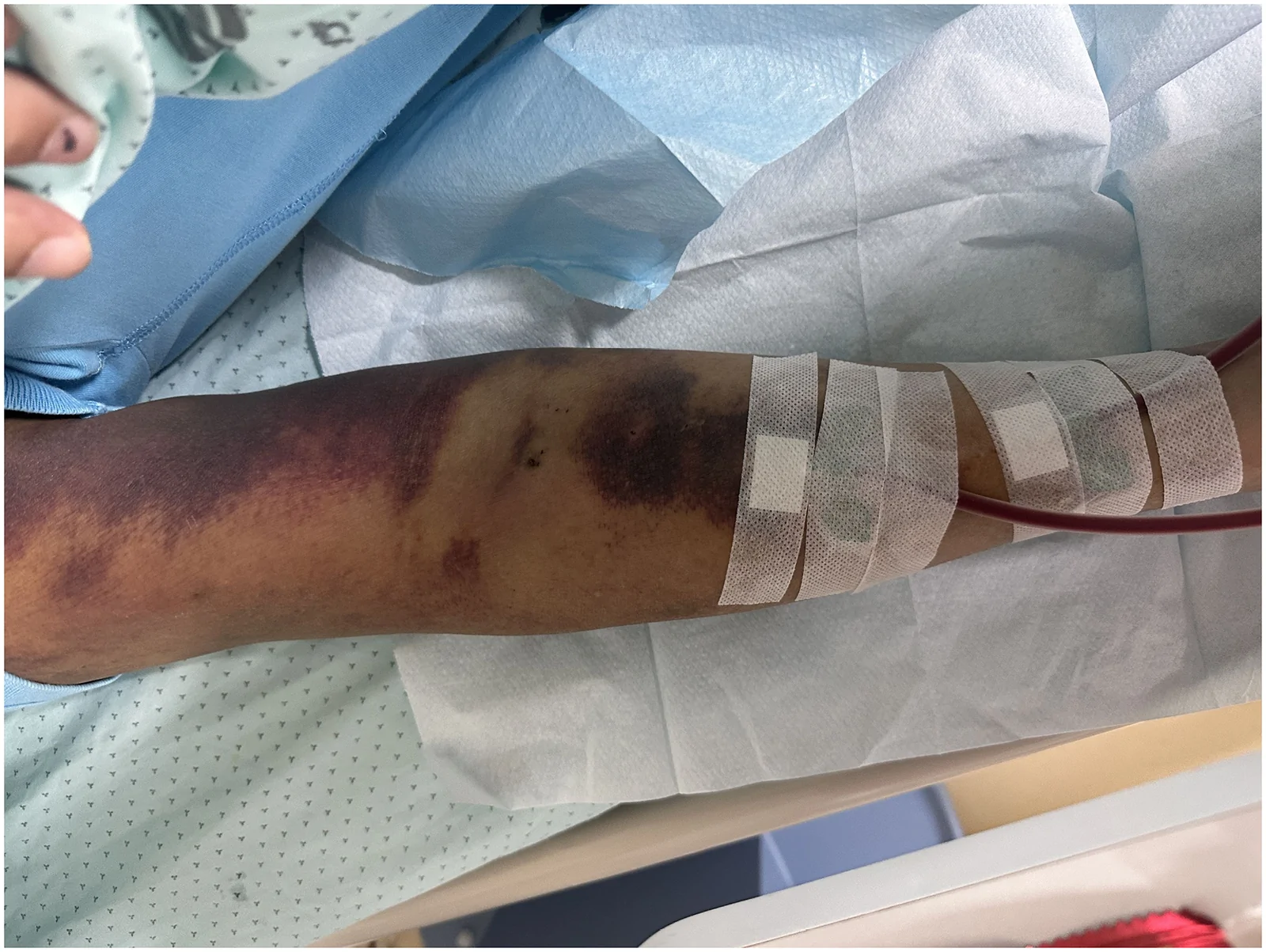
Condition of the patient’s right upper extremity on postoperative day 1.

Routine postoperative care included elevation of the affected limb, close monitoring of distal circulation, and patient education. At 24 hours after the procedure, a structured intervention combining topical application of fresh potato slices with warm moist compresses was initiated. Fresh potatoes were washed, peeled, and cut into slices approximately 0.3 to 0.4 cm thick. The potato slices were applied along the course of the vascular access, beginning 3 to 5 cm proximal to the puncture site and covering the areas with the most pronounced swelling and ecchymosis. The slices were left in place continuously for 2 hours and were replaced immediately if they became dry, oxidized, or discolored to maintain contact with the skin and local moisture. After removal of the potato slices, a warm moist towel maintained at 40°C to 45°C was applied to the same area for 20 to 30 minutes. The combined intervention was administered 3 times daily, with an interval of at least 2 hours between sessions.

Comprehensive nursing care was provided before and after the procedure. Before the procedure, the nursing team conducted a comprehensive assessment of the patient’s general health and major organ function, including cardiac, pulmonary, hepatic, and renal function. The patient’s hemodialysis history and the clinical characteristics of the AVF stenosis were also reviewed. The patient’s psychological status was assessed, and appropriate emotional support and reassurance were provided when needed. After the procedure, postoperative care included close monitoring of vital signs and the vascular access site. Blood flow through the AVF was assessed regularly to evaluate vascular access patency and function. The patient received detailed guidance on postoperative rehabilitation and care. Potential complications, including infection and bleeding, were closely monitored to facilitate prompt management when necessary.

After initiation of the combined intervention, the patient showed progressive improvement in pain, swelling, and ecchymosis. The NRS pain score decreased from 6 to 3, accompanied by visible reductions in swelling and ecchymosis. By postoperative day 4, the NRS pain score had decreased further to 0. Swelling and ecchymosis of the right upper extremity had substantially subsided, and the color of the ecchymosis had changed from purplish red to greenish purple (Fig. 3). Joint mobility had nearly returned to normal, and the patient reported improved overall comfort. By postoperative day 6, the extent of the ecchymosis had further diminished, with its color changing from greenish purple to yellowish green (Fig. 4). Joint mobility had returned to normal, and the AVF could be used for routine cannulation. During the intervention period, the patient reported satisfactory comfort, and no signs of infection were observed at the puncture site. No allergic skin reactions, irritant erythema, or other intervention-related adverse events were observed. In this patient, improvements in pain, swelling, ecchymosis, joint mobility, and comfort were observed during the intervention period, although the independent effect of the combined intervention cannot be determined from a single case.

**Figure 3.**
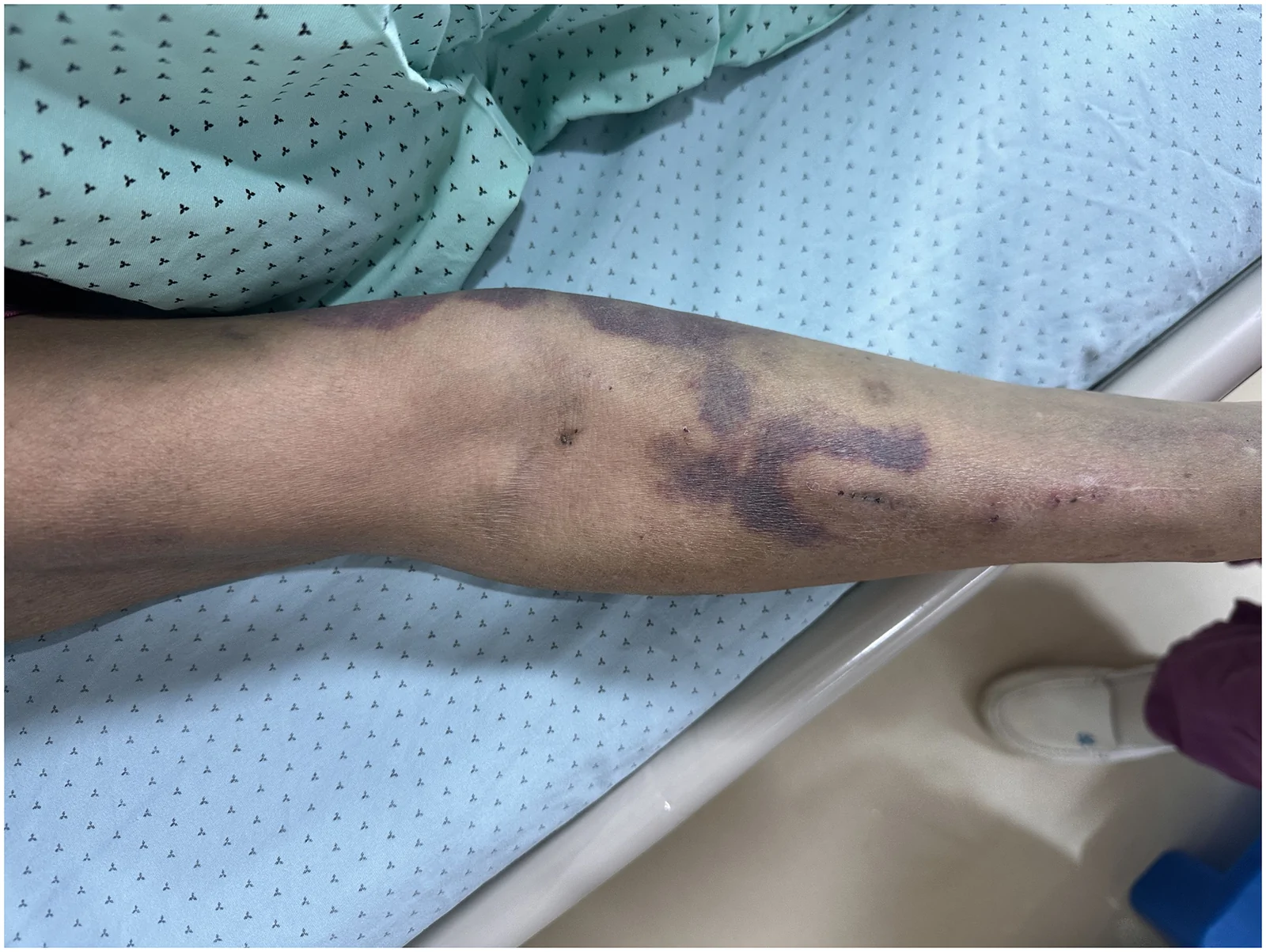
Condition of the patient’s right upper extremity on postoperative day 4.

**Figure 4.**
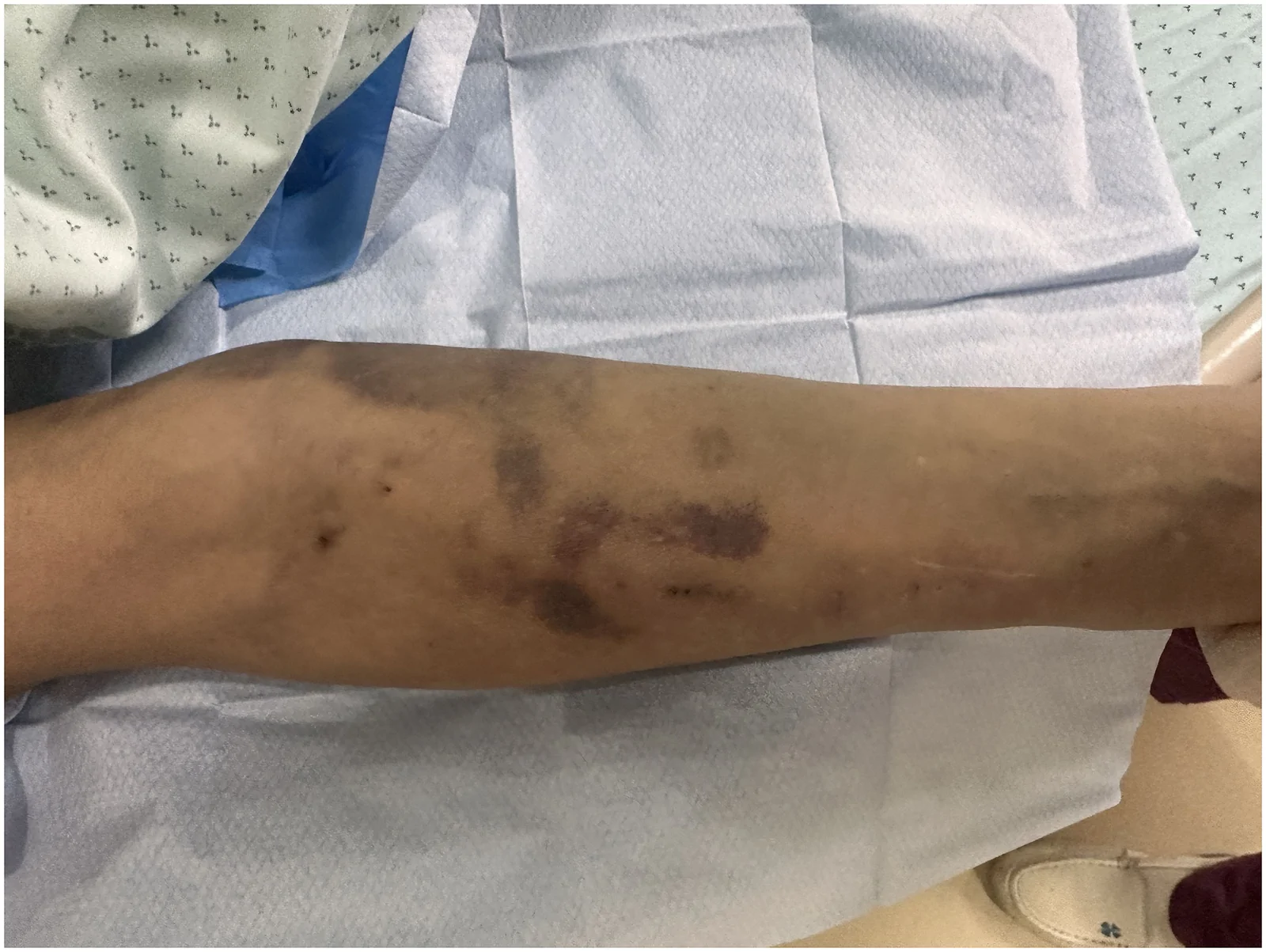
Condition of the patient’s right upper extremity on postoperative day 6.

## 3. Discussion

Endovascular stent placement is widely used as a minimally invasive treatment for AVF stenosis.^[21–25]^ This approach can restore vascular patency and may prolong the functional lifespan of the vascular access. By preserving vascular access function, stent placement can support the continued delivery of MHD. Despite its minimally invasive nature, stent placement may be associated with procedure-related complications. Limb swelling and extensive ecchymosis are clinically important postoperative complications that may be difficult to manage. These complications may cause localized pain and discomfort and may contribute to prolonged hospitalization and increased healthcare costs. Severe limb swelling may hinder assessment and cannulation of the AVF and, in some cases, may compromise the vascular access site. Impaired vascular access function may reduce blood flow and dialysis adequacy and may adversely affect patients’ quality of life. Endovascular interventions should be selected according to the clinical characteristics of the stenosis and the condition of the vascular access. Postoperative care should also emphasize early recognition and management of limb swelling, ecchymosis, and other procedure-related complications. Appropriate postoperative management may help preserve AVF function and facilitate subsequent hemodialysis.

Standardized clinical protocols for managing swelling and ecchymosis after stent placement in an AVF remain lacking. Conventional nursing measures include elevation of the affected limb, warm moist compresses with 50% magnesium sulfate solution, infrared irradiation, and topical medications. Each approach has practical limitations, and evidence supporting its effectiveness in this setting remains limited. Magnesium sulfate wet compresses are thought to reduce tissue edema through an osmotic effect.^[26,27]^ Their effectiveness in resolving established subcutaneous ecchymosis may be limited, and the need for frequent replacement of the compress may also reduce patient adherence. Infrared irradiation may increase local tissue temperature and blood flow through its thermal effects.^[28,29]^ Its use during the acute phase requires caution because excessive heat may aggravate local edema or exudation. In addition, infrared irradiation primarily provides a thermal effect and may not directly address the inflammatory processes associated with tissue injury. Topical anti-inflammatory and analgesic agents may relieve local symptoms but can cause allergic or irritant skin reactions in susceptible patients. Prolonged use may also increase treatment costs and the risk of local adverse effects.

Several plant-derived products have been reported to exhibit anti-inflammatory, analgesic, and edema-reducing activities.^[30–32]^ Sharma et al reviewed the phytochemical and pharmacological properties of *Datura*, a genus within the Solanaceae family.^[33]^ Species of *Datura* contain diverse bioactive constituents and have demonstrated analgesic, antiviral, antidiarrheal, and anti-inflammatory activities in preclinical studies. These findings suggest potential therapeutic applications, although their clinical relevance and safety require further evaluation. Potatoes, which also belong to the Solanaceae family, contain starch, proteins, vitamins, organic acids, minerals, and small amounts of steroidal glycoalkaloids such as solanine.^[21,34,35]^ Some potato-derived constituents have shown antioxidant and anti-inflammatory activities, although evidence for tissue-repair effects after topical application remains limited.^[36]^ Warm moist compresses are commonly used as a physical nursing measure and may increase local tissue temperature and blood flow, thereby facilitating the resolution of edema and ecchymosis. Combining fresh potato slices with warm moist compresses provides a structured nursing intervention, although any synergistic effect remains to be confirmed in controlled studies.

The potential effects of this combined intervention may involve both physical and biochemical processes, although the underlying mechanisms remain uncertain. From a physical perspective, the high water content of fresh potato slices may produce a mild cooling effect when applied topically. This cooling effect may promote transient vasoconstriction and limit local fluid accumulation, potentially reducing early swelling. Alternating topical cooling with warm moist compresses may induce repeated changes in local vascular tone. These temperature changes may influence local blood flow and lymphatic drainage, thereby facilitating the resolution of edema and ecchymosis. From a biochemical perspective, potato-derived constituents may contribute to local effects, although their activity after topical application has not been established. Solanine has demonstrated anti-inflammatory and analgesic activities in experimental studies,^[37–39]^ but its effects and safety after topical application of fresh potato slices remain unclear. Citric acid is present in potatoes, although evidence that topical exposure improves capillary dilation or microcirculation is limited. Vitamin C contributes to collagen synthesis and tissue maintenance, but the extent to which it is absorbed from topical potato slices and influences vascular integrity is unknown. Collectively, these proposed mechanisms may offer a biological rationale for the intervention, but they were not directly evaluated in this patient and require further experimental and clinical investigation.

However, this study has several limitations. First, it is only a single case report. Although the observed improvement was temporally associated with the intervention, the level of evidence was limited, and the contribution of individual patient characteristics or concurrent postoperative nursing care cannot be excluded. Second, the assessment relied mainly on subjective feedback and visual inspection, without objective quantitative measures to evaluate changes in swelling and bruising. These could have included ultrasound evaluation of subcutaneous tissue thickness, precise limb circumference measurements, or assays of serum inflammatory markers. In addition, the lack of a control group precluded direct statistical comparisons and prevented determination of the independent effect of potato compresses. Future prospective studies with larger sample sizes, standardized outcome assessments, and appropriate control groups are warranted to further evaluate the effectiveness of this intervention.

The combination of fresh potato slices and warm moist compresses is inexpensive, simple to administer, and readily accessible. The intervention also requires no specialized equipment. These practical features may make the intervention feasible in resource-limited settings or where access to topical medications is restricted. Because the present report describes only a single patient, the observed improvement cannot establish the effectiveness of the intervention. Further evaluation in well-designed prospective studies is warranted. A prospective randomized controlled trial could evaluate the intervention more rigorously. Such a trial could include conventional care, magnesium sulfate wet compress, infrared irradiation, and combined-intervention groups. Standardized outcomes, including limb circumference, ultrasonographic assessment of tissue edema, pain scores, and adverse events, should be used to compare the groups objectively. Future studies should also determine the optimal frequency and duration of application. Experimental studies are also needed to investigate the biological mechanisms underlying any observed effects. Its applicability to other forms of localized swelling or soft-tissue injury, such as phlebitis, chemotherapy-related extravasation injury, and post-traumatic tissue damage, may also merit investigation. This case provides preliminary practical insight into a simple adjunctive nursing approach that clinicians may consider when conventional supportive measures are insufficient, although broader clinical use requires further evidence.

## 4. Conclusion

This case reports the use of a structured protocol combining fresh potato slices with warm moist compresses to manage limb swelling and ecchymosis after venous stent placement in a patient receiving hemodialysis. This approach is simple, inexpensive, readily available, and easy to implement. Prospective controlled studies using standardized quantitative outcomes are required to evaluate the effectiveness, safety, and clinical applicability of this intervention.

## Author contributions

**Conceptualization:** Qin Zhang, Zhuoyin Wang, Xiaojuan Han, Jiani Bao, Qi Chen.

**Investigation:** Qin Zhang, Xiaojuan Han, Yaru Yang.

**Methodology:** Qin Zhang, Qi Chen.

**Supervision:** Qin Zhang, Zhuoyin Wang, Qi Chen.

**Validation:** Qin Zhang, Hongqun Zhang.

**Writing – original draft:** Qin Zhang.

**Writing – review & editing:** Qin Zhang, Qi Chen.
